# Healthcare resources and differences in kidney disease-related mortality in Italy: a longitudinal study

**DOI:** 10.1007/s40620-025-02452-w

**Published:** 2025-11-05

**Authors:** Angelo d’Errico, Martina Ventura, Luisa Frova, Vincenzo Bellizzi, Alessio Petrelli, Giuseppe Quintaliani, Simone Navarra, Christian Napoli, Giovanni Gambaro, Anteo Di Napoli

**Affiliations:** 1 Epidemiology Unit, ASL TO3, Collegno, Turin, Italy; 2 Epidemiology Unit, National Institute for Health, Migration and Poverty (INMP), Via di S. Gallicano 25/a 00153, Rome, Italy; 3 National Institute of Statistics (Istat), Rome, Italy; 4 Division of Nephrology and Dialysis, Department Medical Sciences, AORN “Sant’Anna E San Sebastiano” Hospital, Caserta, Italy; 5 Unitelma Sapienza, University of Rome, Rome, Italy; 6 Health Directorate, National Institute for Health, Migration and Poverty (INMP), Rome, Italy; 7 Department of Medical Surgical Sciences and Translational Medicine, Sapienza University of Rome, Rome, Italy; 8 Division of Nephrology and Dialysis, Ospedale Maggiore, University of Verona, Verona, Italy

**Keywords:** Kidney disease-related mortality, Healthcare resources, Regional differences, Nationwide data

## Abstract

**Background:**

Significant differences in kidney disease-related mortality persist among Italian regions, even after adjusting for age and education level, suggesting a role of contextual factors. The study aimed to assess whether these differences are attributable to the availability of economic and structural resources for healthcare.

**Methods:**

Retrospective longitudinal cohort study conducted on the Italian population recorded in the 2011 Census and followed up to 2019. Deaths from kidney diseases were retrieved by record linkage with the Causes of Death Register. Regional information on age-adjusted prevalence of kidney disease (indicator of demand for care), current healthcare expenditure per capita, and number of nurses and beds in dialysis units (indicators of renal care supply) per million residents were selected as contextual variables. Regional differences in kidney disease-related mortality taking or not into account these contextual indicators were evaluated using a multilevel approach.

**Results:**

Age-adjusted kidney disease-related mortality rates were higher than the national average for males and females in the largest southern regions. When adding to the models the prevalence of kidney disease, healthcare expenditure, and number of nurses and beds in dialysis units, regional differences in kidney disease-related mortality became non-significant compared to the national average. Significant heterogeneity persisted across regions, both in males and females, although its magnitude strongly decreased when regional-level covariates were considered.

**Conclusions:**

Regional differences in kidney disease-related mortality decreased markedly after considering the general expenditure for healthcare and the number of nurses in dialysis units, suggesting that resources dedicated to caring for kidney disease patients may play an important role in decreasing their mortality.

**Graphical abstract:**

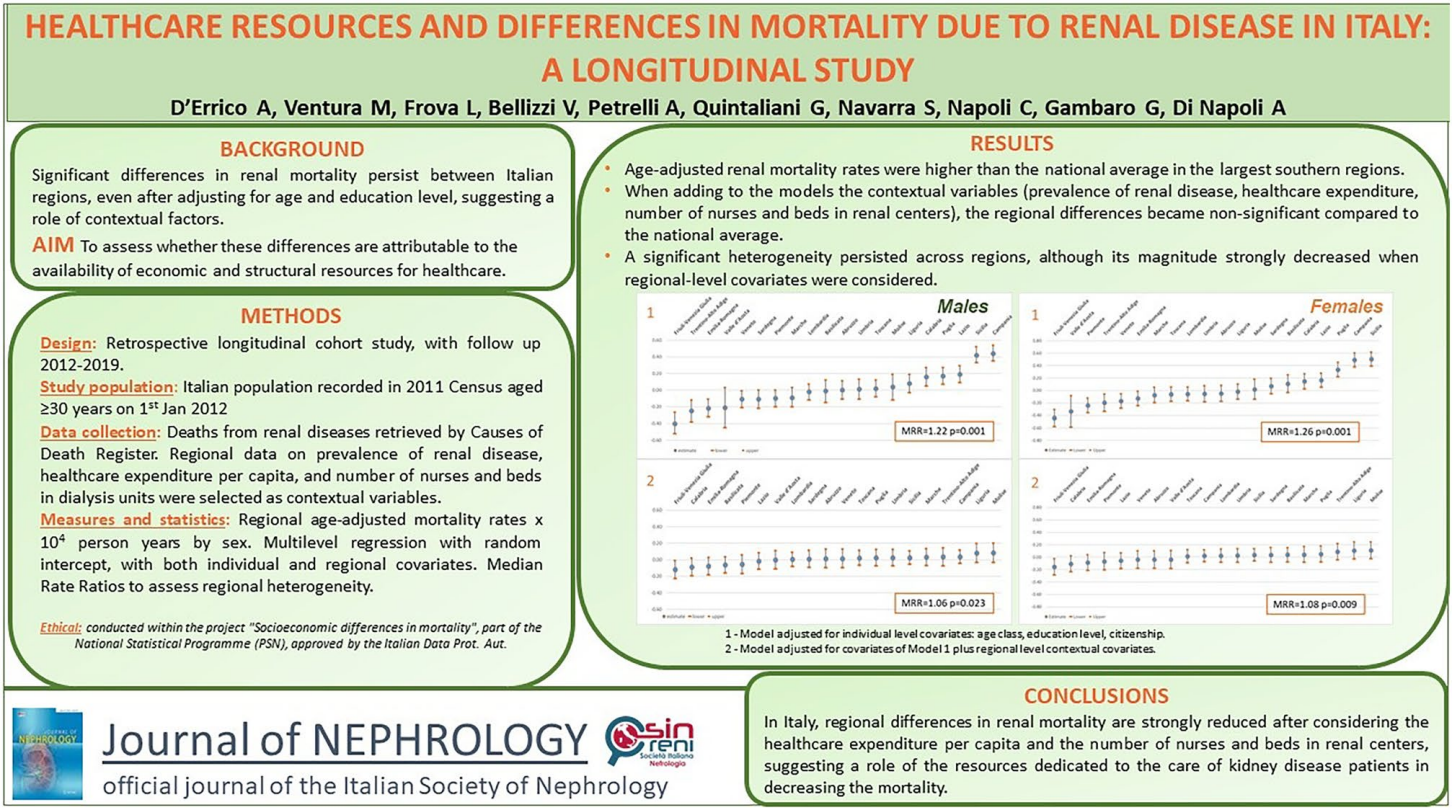

**Supplementary Information:**

The online version contains supplementary material available at 10.1007/s40620-025-02452-w.

## Introduction

Chronic nephropathies are common conditions characterized by the progressive decline in kidney function, possibly leading to kidney failure, and by high cardiovascular and mortality risk [1–3]. In the natural history of nephropathy, several dysfunctions occur, and diabetes and hypertension are the two most frequent causes of chronic nephropathies [4, 5]. Treatment of these dysfunctions and comorbidities requires a long-term comprehensive clinical approach that, together with the costs of kidney replacement therapy, dialysis, and transplantation, results in high costs for healthcare systems [6, 7].

A Global Burden of Disease study has reported geographical differences in age-standardized chronic kidney disease (CKD) incidence, prevalence, and mortality, generally with higher rates in countries characterized by lower socioeconomic development [8–10]. Furthermore, even in areas with similar sociodemographic features, like Western Europe, age-adjusted CKD incidence and mortality rates show a more than two-fold difference across countries [11]. These findings suggest that geographical differences in CKD mortality rates may be explained, at least in part, by differences in access to adequate care during pre-dialysis stages [11, 12].

In Italy, significant differences in mortality due to kidney disease (from now on called “kidney disease-related mortality”) also exist between regions. The highest mortality rates have been observed in southern regions, which are those with the most disadvantaged socioeconomic conditions [13]. These differences may reflect existing differences between regions with regard to the incidence of kidney disease due to the known social gradient of the main risk factors of CKD, such as diabetes or hypertension [14, 15]. However, as significant differences across Italian regions persisted after adjusting for individual education level [13], it seems likely that they may also be attributable to contextual factors, related to the provision of effective treatment to prevent kidney failure.

The Italian National Healthcare System is organized at the regional level, with national funding based primarily on the age distribution of the resident population. In the last two decades, seven Italian regions have been subjected to a prolonged reduction in healthcare funding for budgetary reasons under the supervision of the Ministry of Economy and Finance (*healthcare deficit recovery plan*). This reduction has likely impacted the organization of regional healthcare systems and their ability to deliver care to the resident population. For example, both healthcare personnel turnover (as a result of a hiring freeze) and number of hospital beds were significantly reduced [16].

The aim of the present study was, therefore, to assess whether the observed differences in kidney disease-related mortality between Italian regions may be attributable to the regional availability of economic and structural resources for healthcare, both in terms of general healthcare funding and in the amount of personnel and care facilities specifically dedicated to the diagnosis and treatment of kidney diseases.

## Methods

### Data collection

Using a retrospective longitudinal design, all individuals recorded in the 2011 Census of the Italian Population were followed up for mortality from 2012 to 2019, yielding a maximum of eight years of observation. Date and cause of death were retrieved from the National Causes of Death Register, and data concerning emigration out of the country were obtained from the Resident Population Register, both managed by the Italian National Institute of Statistics (Istat) [17].

All individuals aged at least 30 years on 1 January, 2012 were selected. Deaths from kidney diseases were identified through ICD-10 codes (Supplementary Table S1).

Information on age, sex, education level, citizenship, and region of residence was collected at the 2011 Census of the Italian Population.

As a general indicator of the availability of healthcare resources, we used the 2012 regional healthcare expenditure per capita, retrieved from the Ministry of Economy and Finance [18]. To assess the regional availability of resources specific to renal healthcare, we used the 2014–2015 Italian Society of Nephrology (SIN) census of renal and dialysis units, which provides information on facilities and personnel in each region [19].

To characterize the different regional contexts in terms of demand for healthcare for kidney diseases, we used the sex-specific and age-standardized regional prevalence of self-reported kidney disease, specifically computed for the study from data of the 2013 National Health Interview Survey [20].

Sex-specific regional CKD prevalence was estimated as the proportion of subjects reporting kidney failure diagnosed by a physician, stratified by sex and standardized by 5-year age class.

### Data analysis

The study population's demographic characteristics and kidney disease deaths are described through frequency distributions.

For reasons related to privacy regulations, the age data made available by Istat for the study were not continuous but aggregated by 5-year age class, that we further categorized into four classes (30–64, 65–74, 75–84, 85+ years). Education level was classified into four classes (university degree, high school, middle school, and elementary school or less), while, based on citizenship, subjects were divided into Italians, migrants from highly developed countries (HDCs), and migrants from high migratory pressure countries (HMPCs).

Regional age-adjusted mortality rates × 1000 person-years were calculated by sex.

To evaluate differences among Italian regions in terms of kidney disease-related mortality, considering both individual characteristics and regional indicators, a multilevel approach was used that took into account the hierarchical structure of the data. We performed multilevel Poisson regression models with random intercept, in which individuals and regions were considered as first- and second-level units, respectively. The first-level covariates were age class, education level, and citizenship.

Among second-level covariates, the sex-specific and age-standardized regional prevalence of self-reported kidney disease (continuous) was included as the regional indicator of demand for renal healthcare. As the regional general indicator of healthcare supply, we included the regional percentage deviation from the national average in current healthcare expenditure per capita (in quintiles). In a preliminary analysis regarding indicators of regional availability of resources specific to renal healthcare, we explored the association between CKD mortality and the available indicators (number of beds and hospital admissions in nephrology departments, and number of doctors, nurses and beds in dialysis units per million population) through multilevel Poisson regression models with random intercept, stratified by sex and adjusted for age, education level and citizenship. Among these indicators, the number of regional nurses and of beds in dialysis units per million population (in quartiles) resulted significantly associated with CKD mortality and, therefore, considered in further multilevel analyses.

Subsequently, we performed different multilevel random intercept models progressively adjusted for indicators of supply and demand of renal care at the regional level, assessing whether differences in kidney disease-related mortality between Italian regions attenuated after taking into account regional differences in these indicators. In Model 1, regional differences in kidney disease-related mortality were evaluated by including only covariates available at the individual level (age class, education level, citizenship). In Model 2, the regional prevalence of self-reported kidney diseases was added to check for differences in renal healthcare demand. In Model 3, the regional healthcare expenditure per capita was also added. Finally, in Model 4, the number of nurses and beds in dialysis units was included. All models were stratified by sex.

Fixed predictor coefficients are reported as cumulative incidence rate ratios (IRRs) of mortality with 95% confidence intervals (CI). Predicted post-estimation counts with 95% CI by region were used to evaluate the geographical differences as regional residuals around first-level intercept, which represents the national mean effect after adjusting for all first- and second-level covariates. As a measure of the magnitude of regional heterogeneity, median rate ratios (MedRRs) were calculated to quantify the general contextual effect. MedRRs describe the median relative change in the kidney disease-related mortality rate when comparing identical subjects from two different randomly selected regions that are ordered by rate [21].

Statistical analyses were performed using SAS 9.3 and STATA 15.

## Results

### Descriptive analysis

The characteristics of the cohort are shown in Supplementary Table S2: more than 50% had a middle-low education level, 30% were aged 65+ years, and foreigners made up 5.4% of the total.

The age-adjusted kidney disease-related mortality rates (× 1000 person-years) by region and sex are shown in Supplementary Fig. 1. A geographical north–south trend was observed, with rates higher than the national average for both sexes in all southern regions.

Supplementary Table S3 summarizes the values of the indicators of healthcare supply and demand included in the multilevel models, by region. Regional sex-specific age-standardized CKD prevalence showed a wide variability, ranging between 0.6% in Molise to almost 2% in Abruzzo and Sardegna among men, and between 0.7% in Trentino-Alto Adige to 1.9% in Calabria among women. According to the SIN 2014–15 Census, large variability was also observed with regard to the number of nurses, ranging between 69 in Lazio to 277 in Sardegna. The current healthcare expenditure per capita in 2012 ranged from 1,670€ in Campania to 2,010€ in Emilia-Romagna.

### Differences in kidney disease-related mortality across Italian regions

**Fig. 1:**
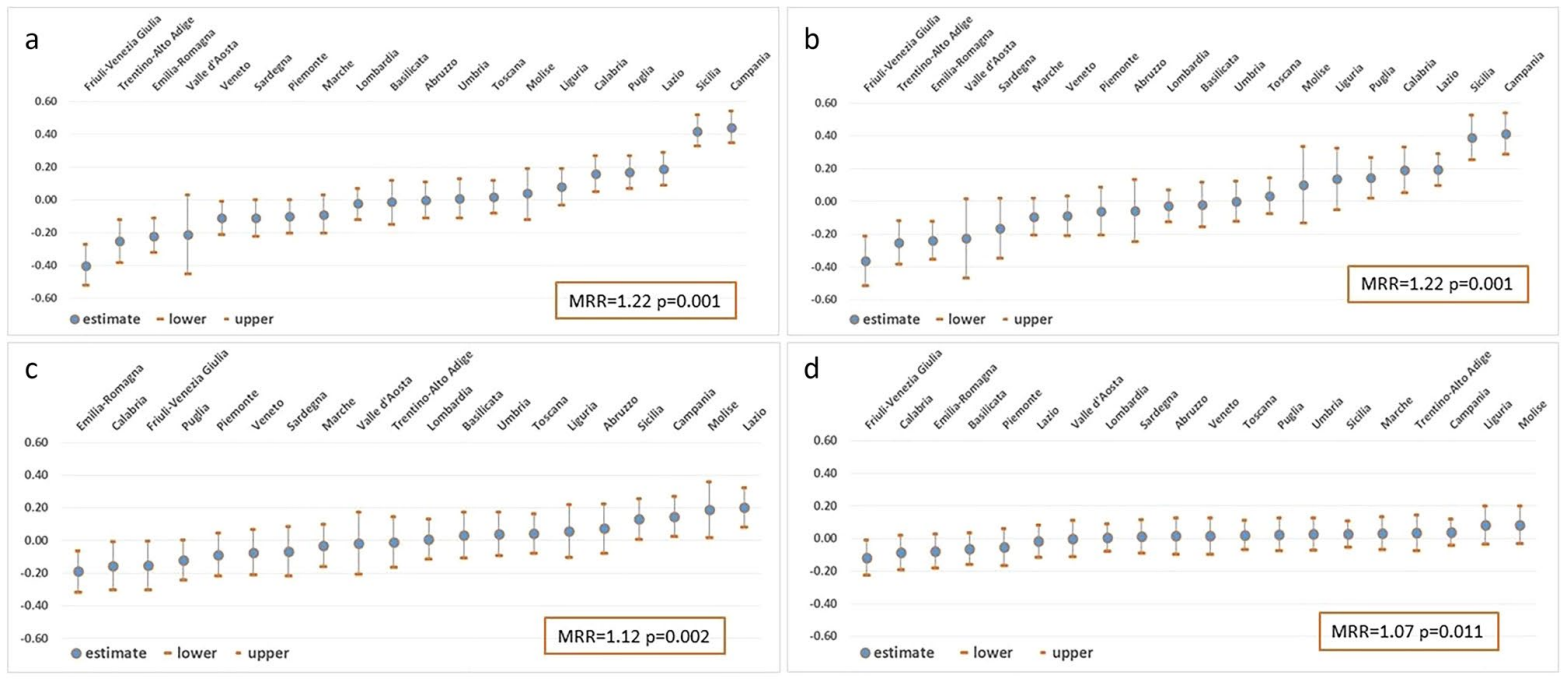
**a**–**d** Net effect of the region as second-level variable: predicted post-estimation counts by region, ranked from the lowest to the highest, and regional median rate ratios (MedRR) from Models 1–4. Males. **a** Model 1 (adjusted for individual level covariates: age class, education level, citizenship); **b** Model 2 (adjusted for covariates of Model 1 plus regional level covariate “prevalence of self-reported kidney diseases”); **c** Model 3 (adjusted for covariates of Model 2 plus regional level covariate “health expenditure per capita”); **d** Model 4 (adjusted for covariates of Model 3 plus regional level covariates “number of nurses and beds” in dialysis units). Net-effect: regional differences around the national mean (average mortality), after adjusting for all the covariates considered at the first-level and second-level

**Fig. 2:**
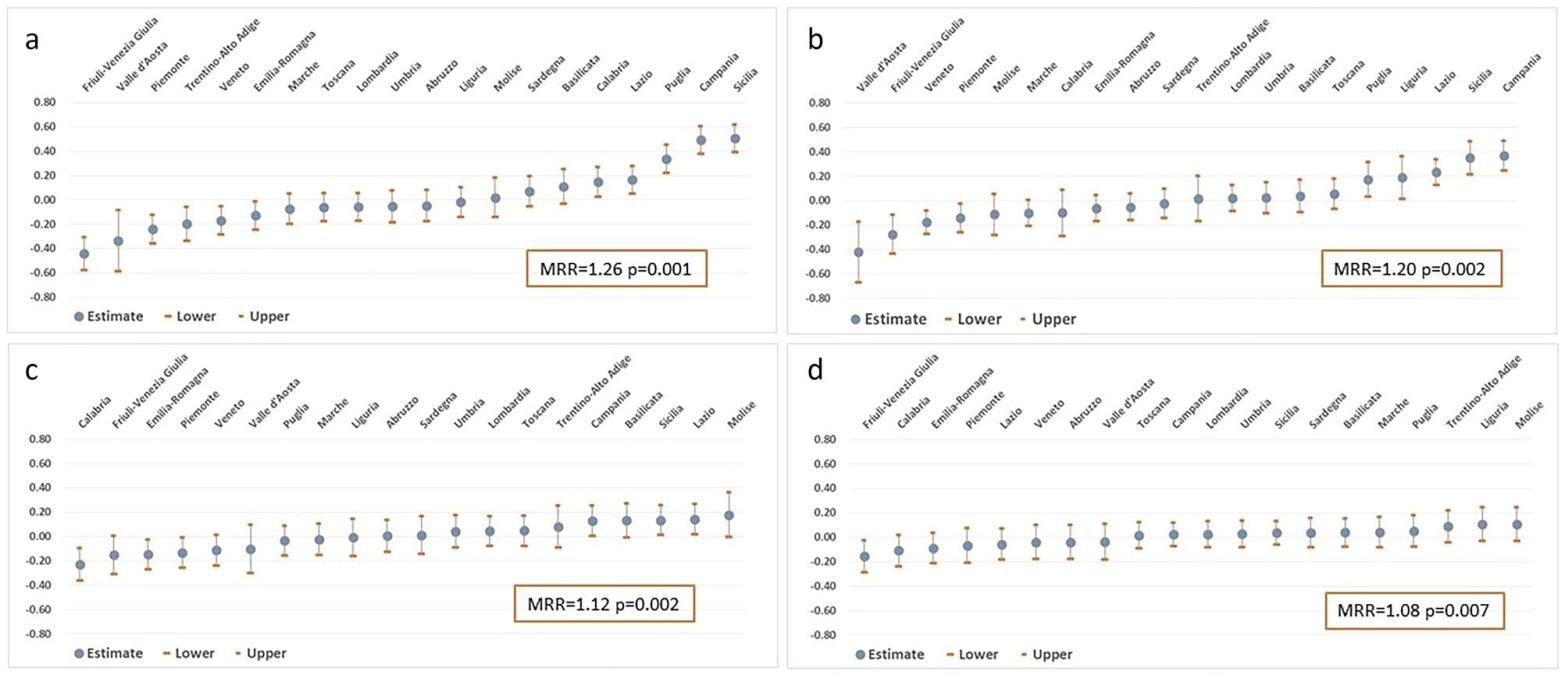
**a**–**d** Net effect of the region as second-level variable: predicted post-estimation counts by region, ranked from the lowest to the highest, and regional median rate ratios (MedRR) from Models 1–4. Females. **a** Model 1 (adjusted for individual level covariates: age class, education level, citizenship); **b** Model 2 (adjusted for covariates of Model 1 plus regional level covariate “prevalence of self-reported kidney diseases”); **c** Model 3 (adjusted for covariates of Model 2 plus regional level covariate “health expenditure per capita”); **d** Model 4 (adjusted for covariates of Model 3 plus regional level covariates “number of nurses and beds” in dialysis units). Net-effect: regional differences around the national mean (average mortality), after adjusting for all the covariates considered at the first-level and second-level

The findings of Models 1–4, starting from the model with only individual covariates to the fully-adjusted one, are presented in Tables 1 and 2 (results for fixed effects), in Figs. 1 and 2 and in Supplementary Tables S4-S5 (results for random effects), for males and females. In Model 1, kidney disease-related mortality showed a strong inverse educational gradient in both sexes (RRadj = 1.54 among males and RRadj = 1.92 among females, comparing the lowest vs. the highest education level) and a significantly lower risk for immigrants from high migratory pressure countries, compared to Italians (RRadj = 0.37).

**Table 1:** Mortality for CKD. Findings of multilevel analyses (fixed effects) with individual and regional covariates (models 1–4)—males

Variables		Model 1			Model 2			Model 3			Model 4		
		RR	95% CI	*P*	RR	95% CI	*P*	RR	95% CI	*P*	RR	95% CI	*P*
Individual covariates													
Education level	Elementary school or less	1.54	1.48–1.60	< 0.001	1.54	1.48–1.60	< 0.001	1.54	1.48–1.60	< 0.001	1.54	1.48–1.60	< 0.001
	Middle school	1.27	1.23–1.31	< 0.001	1.27	1.23–1.31	< 0.001	1.27	1.23–1.31	< 0.001	1.27	1.23–1.31	< 0.001
	High school diploma	1.12	1.08–1.16	< 0.001	1.12	1.08–1.16	< 0.001	1.12	1.08–1.16	< 0.001	1.12	1.08–1.16	< 0.001
	University degree or more	1.00	–		1	–	–	1	–	–	1	–	–
Age class	30–64	1.00	–		1	–	–	1	–	–	1	–	–
	65–74	7.95	7.51–8.42	< 0.001	7.95	7.51–8.42	< 0.001	7.95	7.51–8.42	< 0.001	7.95	7.51–8.42	< 0.001
	75–84	34.17	32.45–35.99	< 0.001	34.17	32.45–35.99	< 0.001	34.18	32.45–36.00	< 0.001	34.17	32.45–35.99	< 0.001
	85+	153.35	145.68–161.43	< 0.001	153.36	145.69–161.43	< 0.001	153.40	145.73–161.48	< 0.001	153.39	145.72–161.46	< 0.001
Citizenship	High migratory pressure countries	0.37	0.30–0.45	< 0.001	0.37	0.30–0.45	< 0.001	0.37	0.30–0.45	< 0.001	0.37	0.30–0.45	< 0.001
	Highly developed countries	0.40	0.27–0.58	< 0.001	0.40	0.28–0.59	< 0.001	0.40	0.28–0.59	< 0.001	0.40	0.27–0.58	< 0.001
	Italian	1.00	–		1	–	–	1	–	–	1	–	–
Regional covariates													
Prevalence of CKD	1% increase				1.10	0.86–1.41	0.46	0.95	0.81–1.13	0.576	0.88	0.76–1.01	0.066
Health care expenditure	Q5 (> 9.50)							1	–		1	–	
	Q4 (4.45–9.50)							1.27	1.05–1.54	0.012	1.25	1.08–1.45	0.004
	Q3 (-0.86; 4.44)							1.25	1.04–1.5	0.017	1.24	1.07–1.45	0.005
	Q2 (-4.60; -0.87)							1.22	1.01–1.48	0.04	1.12	0.96–1.32	0.160
	Q1 (< -4.60)							1.76	1.45–2.13	< 0.001	1.58	1.35–1.85	< 0.001
Nurses in dialysis units	Q1 (< 126)										1.31	1.06–1.62	0.011
	Q2 (126–200)										1.00	0.85–1.19	0.973
	Q3 (201–216)										1.01	0.87–1.18	0.878
	Q4 (> 216)										1	–	
Beds in dialysis units	Q1 (< 157)										0.99	0.78–1.25	0.932
	Q2 (157–179)										0.95	0.8–1.12	0.525
	Q3 (180–210)										1.03	0.85–1.23	0.793
	Q4 (> 210)										1	–	–

*RR* Rate Ratio, *CI* Confidence Interval

**Table 2:** Mortality for CKD. Findings of multilevel analyses (fixed effects) with individual and regional covariates (models 1–4)—females

Variables		Model 1			Model 2			Model 3			Model 4		
		RR	95% CI	*P*	RR	95% CI	*P*	RR	95% CI	*P*	RR	95% CI	*P*
Individual covariates													
Education level	Elementary school or less	1.92	1.85–2.00	< 0.001	1.92	1.85–2.00	< 0.001	1.92	1.85–2.00	< 0.001	1.92	1.85–2.00	< 0.001
	Middle school	1.55	1.49–1.61	< 0.001	1.55	1.49–1.61	< 0.001	1.55	1.49–1.61	< 0.001	1.55	1.49–1.61	< 0.001
	High school diploma	1.22	1.16–1.27	< 0.001	1.22	1.16–1.27	< 0.001	1.22	1.16–1.27	< 0.001	1.22	1.16–1.27	< 0.001
	University degree or more	1	–		1	–	–	1	–	–	1	–	–
Age class	30–64	1	–		1	–	–	1	–	–	1	–	–
	65–74	7.36	6.85–7.91	< 0.0001	7.36	6.86–7.91	< 0.001	7.36	6.85–7.91	< 0.001	7.36	6.85–7.91	< 0.001
	75–84	32.15	30.14–34.30	< 0.0001	32.16	30.15–34.31	< 0.001	32.16	30.15–34.30	< 0.001	32.16	30.15–34.30	< 0.001
	85+	153.01	143.62–163.00	< 0.0001	153.06	143.67–163.06	< 0.001	153.05	143.66–163.05	< 0.001	153.06	143.68–163.06	< 0.001
Citizenship	High migratory pressure countries	0.37	0.30–0.46	< 0.0001	0.37	0.30–0.46	< 0.001	0.37	0.30–0.46	< 0.001	0.37	0.30–0.46	< 0.001
	Highly developed countries	0.58	0.41–0.83	< 0.0001	0.58	0.41–0.82	0.002	0.58	0.41–0.83	0.003	0.58	0.41–0.83	0.003
	Italian	1	–		1	–	–	1	–	–	1	–	–
Regional covariates													
Prevalence of CKD	1% increase				1.46	1.14–1.86	0.003	1.09	0.87–1.37	0.458	0.96	0.72–1.29	0.793
Health care expenditure	Q5 (> 9.50)							1	–		1	–	
	Q4 (4.45–9.50)							1.33	1.11–1.59	0.002	1.36	1.12–1.64	0.002
	Q3 (− 0.86; 4.44)							1.18	0.98–1.41	0.081	1.19	1–1.41	0.045
	Q2 (− 4.60; − 0.87)							1.21	1–1.46	0.049	1.17	0.94–1.45	0.166
	Q1 (< − 4.60)							1.79	1.43–2.24	< 0.001	1.77	1.33–2.35	< .0001
Nurses in dialysis units	Q1 (< 126)										1.37	1.08–1.75	0.011
	Q2 (126–200)										1.13	0.91–1.41	0.280
	Q3 (201–216)										1.12	0.88–1.43	0.350
	Q4 (> 216)										1	–	
Beds in dialysis units	Q1 (< 157)										0.87	0.65–1.18	0.372
	Q2 (157–179)										0.82	0.62–1.08	0.152
	Q3 (180–210)										0.89	0.68–1.16	0.387
	Q4 (> 210)										1	–	

*RR* Rate Ratio, *CI* Confidence Interval

When adding the regional prevalence of self-reported kidney disease to individual covariates (model 2), a significant association with kidney disease-related mortality was only observed in females (RRadj = 1.46 for a 1% increase in regional prevalence).

In Model 3, the regional healthcare expenditure was strongly associated with kidney disease-related mortality in males and females (RRadj = 1.76 and RRadj = 1.79 for the lowest vs. the highest quintile, respectively).

When including the regional number of nurses in dialysis units (model 4), a higher risk of kidney disease-related mortality was observed for the lowest quartile of the distribution of the number of nurses, both in males and females (RRadj = 1.31 and RRadj = 1.37, respectively). In contrast, the number of beds in dialysis units was not associated with mortality. Furthermore, the association of kidney disease-related mortality with regional healthcare expenditure was slightly attenuated among males in this model, while remaining unchanged among females.

The predicted net effect with 95% CI of the region as a second-level variable, holding fixed the first-level factors, is shown in Fig. 1a–d (Supplementary Table S4) for males and Fig. 2a–d (Supplementary Table S5) for females, ranked from the lowest to the highest.

In Model 1, eight regions resulted significantly different from zero (Fig. 1a): five with kidney disease-related mortality higher than the national average (Calabria, Lazio, Puglia, Campania, and Sicilia) and three with lower mortality (Emilia-Romagna, Friuli-Venezia Giulia, Trentino-Alto Adige). Even after adjusting the models for the regional prevalence of self-reported kidney disease, the results did not change substantially (Fig. 1b). Instead, when taking into account the regional healthcare expenditure per capita (Fig. 1c), differences among the regions attenuated. However, significantly higher kidney disease-related mortality was confirmed for Campania, Sicilia, and Lazio, while Molise also emerged as a region with significantly higher mortality. Emilia-Romagna and Friuli-Venezia Giulia maintained a significantly lower risk, together with Calabria, for which the direction of the effect changed compared to Model 1. When considering all the supply and demand covariates, the predicted net effect was still significantly lower than the national average only for Friuli-Venezia Giulia (Fig. 1d).

Among females, in the analysis adjusted for individual covariates (Fig. 2a), the same five regions observed for males (Calabria, Lazio, Puglia, Campania, and Sicilia) had significantly higher mortality than the national average, and with the same ranking. Furthermore, six regions showed a significantly lower risk (Friuli-Venezia Giulia, Valle d’Aosta, Piemonte, Trentino-Alto Adige, Veneto, and Emilia-Romagna). As was the case for males, higher kidney disease-related mortality persisted after accounting for regional self-reported kidney disease prevalence in four out of five regions, with Liguria added to the list and Calabria now not differing from the national mean (Fig. 2b). A substantial decrease in regional differences was observed after adjusting for differences in healthcare expenditure (Fig. 2c), with only Campania, Sicilia, and Lazio still showing a significantly higher effect, and Emilia-Romagna, Piemonte, and Calabria having a significantly lower one. By also including the number of nurses and beds in dialysis units per million residents in each region (Fig. 2d) among the second-level covariates, the regional differences in kidney disease-related mortality became minimal, as it did in males, with only Friuli-Venezia Giulia showing a significant advantage compared to the national mean.

The MedRRs were statistically significant for all four models, both in males (Fig. 1a–d) and in females (Fig. 2a–d), confirming the presence of heterogeneity among regions. However, in both sexes, the magnitude of this heterogeneity strongly decreased from Model 1 to Model 4, suggesting that the general contextual effect may be partially explained by the variables added to the models.

## Discussion

In this population-wide study, we found important differences in kidney disease-related mortality across Italian regions, even after accounting for individual sociodemographic covariates, with the largest southern regions (Campania, Sicilia, Puglia, Calabria) at greater risk in both sexes. These four regions were the ones with the lowest gross domestic product per capita in the country in the last decades [22]. In contrast, four regions showed significantly lower kidney disease-related mortality than the average (Emilia-Romagna, Friuli-Venezia Giulia, Trentino-Alto Adige, Veneto), all located in the North and among the wealthiest areas of Italy.

Adjusting for the regional prevalence of self-reported kidney disease slightly impacted the associations, whereas taking into account differences in regional healthcare funding produced substantial attenuation of their strength in both sexes, particularly for Campania and Sicilia, the regions with the highest kidney disease-related mortality. The adjustment for the regional number of nurses and beds in dialysis units resulted in a further attenuation of the RRs and a loss of their statistical significance for all regions except for Friuli-Venezia Giulia. These results suggest that regional differences in the availability of healthcare resources, in terms of both general expenditure and size of dialysis units, in terms of beds and nursing personnel, are factors that could explain the regional differences in kidney disease-related mortality across Italian regions. The number of nurses per capita clearly may affect the quality of care of kidney disease patients, thus impacting the natural history of the disease and mortality. It is worth considering that the five regions (Campania, Sicilia, Lazio, Calabria, Puglia) found to be at higher risk in the analysis adjusted only for individual sociodemographic covariates (Model 1) are among the seven regions that are still under the *healthcare deficit recovery plan*, a national economic program limiting their healthcare expenditure [23]. Their healthcare funding per capita in the last years is still 15–20% lower than the rest of the country and approximately 30% lower than the northern regions (which include all regions found to be at significantly lower risk of kidney disease-related mortality). It is also important to mention that the southern regions, compared to those in the North, started from a lower funding base due to their populations' age structure being younger, the main criterion used to allocate financial resources among regions.

In the five regions with the highest kidney disease-related mortality, the *healthcare deficit recovery plan* has been ongoing for over a decade (it started in 2007 for Campania, Sicilia, and Lazio, in 2009 for Calabria, and in 2010 for Puglia), covering the entire observational period of this study. Therefore, such a long-term reduction in the regional resources for healthcare may have impacted the adequate delivery of care to renal patients and may have resulted in higher kidney disease-related mortality. This possibility appears strengthened by the finding that the size of the nursing personnel in dialysis units in each region contributed to explaining regional differences in kidney disease-related mortality even after considering regional differences in healthcare funding. Of note, the number of nurses in dialysis units per million residents showed significant variability across regions, with the lowest values being observed for regions with the highest kidney disease-related mortality, particularly Campania, Sicilia and Lazio, where they were less than half the national average.

Kidney diseases represent a major public health challenge because their economic burden on the healthcare system is substantial [8, 24]. Although most of the costs per patient in the renal population are related to kidney failure, earlier and less severe stages also generate financial costs and impact on the death rate and kidney failure incidence [25, 26]. It is evident, therefore, how political decisions, like the *healthcare deficit recovery plan*, have a very short horizon, saving money in the short term but leaving a negative long-term health and budgetary legacy.

Several studies in the USA, Canada, and Norway, have demonstrated that having access to good-quality pre-dialytic care substantially impacts mortality, thus partially explaining geographic differences between less and more socially and economically deprived areas, which may also depend on the healthcare system organization (private insurance vs. public universal healthcare system, varying levels of coverage) [27–31]. Our study supports these findings and highlights the disadvantages that can persist even within a public universal healthcare system like the Italian one. A primary strength of the study is that it was conducted on the whole Italian population aged 30 years or over (more than 40 million people). Furthermore, the large population gave the study great statistical power to examine differences in kidney disease-related mortality by region, also stratifying by sex, thanks to the long follow-up covering almost a decade.

Access to various data sources provided a key advantage for this study, as it allowed for the availability of a large set of data (individual, organizational, administrative, economic) that is rarely found in other studies, enabling adjustments for a wide range of variables.

Another strength is that the regional indicators of healthcare that were used (general healthcare expenditure, number of nurses in dialysis units per million residents) were derived from administrative data. Therefore, they can be considered objective measures, unlikely influenced by potential information bias.

Furthermore, while there are numerous studies analyzing the negative effects of patient-centered variables (i.e. low personal income, comorbidities, marital status, racial differences, service connection) on renal insufficiency [32], our study has added the healthcare system characteristics to the analysis, testing their impact on kidney disease-related mortality.

The study's main limitation is the potential ecological bias related to the variables measured at a regional level. In fact, other regional characteristics that are correlated with general healthcare supply, and potentially associated with kidney disease-related mortality, could explain the attenuation of differences in kidney disease-related mortality, for example the accessibility to treatment for kidney diseases or the capability of healthcare operators to provide effective treatment. Furthermore, information on the regional prevalence of CKD was estimated from self-reported information, which may not be accurate, given that the majority of affected individuals are unaware of the disease and often are diagnosed after several years [33]. Moreover, possible differences in CKD awareness between Italian regions could have affected the differences in the estimates of regional CKD prevalence. Although this issue has never been investigated, it could contribute to explaining the lack of association between CKD prevalence and CKD mortality in fully adjusted models and its small impact on regional differences in CKD mortality.

Last, the lack of awareness has been reported among CKD patients also in the presence of diabetes or hypertension, with a consequent underestimation of mortality caused by CKD and an overestimation of mortality due to these disorders [33]. It appears however difficult to evaluate whether there are differences in the extent of misclassification of deaths due to CKD across regions and if they could have affected our results. Nonetheless, even though we cannot measure the difference in awareness between residents of different regions, we can assume that the level of education included in the models may represent a proxy for cultural differences.

## Conclusions

Regional differences in kidney disease-related mortality are strongly reduced after considering the general healthcare expenditure per capita and the number of nurses and beds in dialysis units per million residents. This finding indicates that such differences may be at least partially attributable to differences in the availability of resources dedicated to the care of kidney disease patients. Given the high costs of treating patients with kidney failure, regional healthcare systems are expected to benefit economically from implementing programs aimed at identifying renal patients in the early stages of the disease to limit the high mortality of kidney disease through adequate treatment.

## Supplementary Information

Below is the link to the electronic supplementary material.

Supplementary file1 (DOCX 270 KB)

## Data Availability

The datasets presented in this article are not readily available. All findings were produced using aggregated and not individual data. Requests to access these datasets should be directed to Istat (www.istat.it).
