# Supplementary material for: Healthcare resources and differences in kidney disease-related mortality in Italy: a longitudinal study
Source: J Nephrol. 2025 Nov 5;38(9):2743–53. doi: 10.1007/s40620-025-02452-w (PMC12712093; doi:10.1007/s40620-025-02452-w)
Supplement: Supplementary file 1 — Supplementary file1 (DOCX 270 KB) [file 40620_2025_2452_MOESM1_ESM.docx]

**SUPPLEMENTARY MATERIALS**

**Supplementary Table S1** - Causes of death considered to identify renal disease mortality (ICD-10)

| Glomerular diseases (N00-N08) | N00 - Acute nephritic syndrome |
| --- | --- |
|  | N01 - Rapidly progressive nephritic syndrome |
|  | N02 - Recurrent and persistent haematuria |
|  | N03 - Chronic nephritic syndrome |
|  | N04 - Nephrotic syndrome |
|  | N05 - Unspecified nephritic syndrome |
|  | N06 - Isolated proteinuria with specified morphological lesion |
|  | N07 - Hereditary nephropathy, not elsewhere classified |
| Renal tubulo-interstitial diseases (N10-N16) | N10 - Acute tubulo-interstitial nephritis |
|  | N11 - Chronic tubulo-interstitial nephritis |
|  | N12 - Tubulo-interstitial nephritis, not specified as acute or chronic |
|  | N13 - Obstructive and reflux uropathy |
|  | N14 - Drug- and heavy-metal-induced tubulo-interstitial and tubular conditions |
|  | N15 - Other renal tubulo-interstitial diseases |
| Renal failure (N17-N19) | N17 - Acute renal failure |
|  | N18 - Chronic kidney disease |
|  | N19 - Unspecified kidney failure |
| Other disorders of kidney and ureter (N25-N29) | N25 - Disorders resulting from impaired renal tubular function |
|  | N26 - Unspecified contracted kidney |
|  | N27 - Small kidney of unknown cause |
|  | N28 - Other disorders of kidney and ureter, not elsewhere classified |

**Supplementary Table 2. Baseline characteristics of the cohort and of deaths (30+ years old)**

|  | **Population at 01-01-2012** | **%** | **Deaths** | **%** |
| --- | --- | --- | --- | --- |
| **Total (Italy)** | 41,878,443 | 100.0 | 71,219 | 100.0 |
| Sex | | | | |
| Males | 19,768,472 | 47.2 | 32,991 | 46.3 |
| Females | 22,109,971 | 52.8 | 38,228 | 53.7 |
| Education level |  |  |  |  |
| Elementary school or less | 11,566,589 | 27.6 | 15,930 | 22.4 |
| Middle school | 12,403,431 | 29.6 | 35,245 | 49.5 |
| High school diploma | 12,693,672 | 30.3 | 10,241 | 14.4 |
| University Degree or more | 5,214,751 | 12.5 | 9,803 | 13.8 |
| Age class | | | | |
| 30-64 | 29,456,791 | 70.3 | 2,917 | 4.1 |
| 65-74 | 6,251,668 | 14.9 | 6,371 | 8.9 |
| 75-84 | 4,467,301 | 10.7 | 21,203 | 29.8 |
| 85+ | 1,702,683 | 4.1 | 40,728 | 57.2 |
| Citizenship | | | | |
| Italian | 39,588,657 | 94.5 | 70,982 | 99.7 |
| High Migratory Pressure Countries | 2,149,659 | 5.1 | 179 | 0.3 |
| Highly Developed Countries | 140,127 | 0.3 | 58 | 0.1 |
| Region | | | | |
| Piemonte | 3,204,126 | 7.7 | 4,475 | 6.3 |
| Valle d'Aosta | 91,378 | 0.2 | 96 | 0.1 |
| Lombardia | 6,934,667 | 16.6 | 9,836 | 13.8 |
| Trentino-Alto Adige | 699,403 | 1.7 | 815 | 1.1 |
| Veneto | 3,461,785 | 8.3 | 4,565 | 6.4 |
| Friuli-Venezia Giulia | 903,980 | 2.2 | 966 | 1.4 |
| Liguria | 1,192,942 | 2.8 | 2,312 | 3.2 |
| Emilia-Romagna | 3,181,755 | 7.6 | 4,648 | 6.5 |
| Toscana | 2,704,531 | 6.5 | 4,718 | 6.6 |
| Umbria | 641,059 | 1.5 | 1,176 | 1.7 |
| Marche | 1,108,426 | 2.6 | 1,882 | 2.6 |
| Lazio | 3,905,982 | 9.3 | 6,829 | 9.6 |
| Abruzzo | 928,326 | 2.2 | 1,606 | 2.3 |
| Molise | 222,490 | 0.5 | 441 | 0.6 |
| Campania | 3,724,079 | 8.9 | 7,935 | 11.1 |
| Puglia | 2,741,841 | 6.5 | 5,612 | 7.9 |
| Basilicata | 399,672 | 1.0 | 768 | 1.1 |
| Calabria | 1,314,388 | 3.1 | 2,565 | 3.6 |
| Sicilia | 3,337,352 | 8.0 | 8,169 | 11.5 |
| Sardegna | 1,180,261 | 2.8 | 1,805 | 2.5 |

| **Supplementary Table 3 - Indicators of healthcare demand and offer, by region** | | | | | |
| --- | --- | --- | --- | --- | --- |
| *REGION* | *Prevalence of individuals who reported having*  *had a diagnosis*  *of CKD (% men) ** | *Prevalence of individuals who reported having*  *had a diagnosis*  *of CKD (% women) ** | *Number of*  *nurses in*  *dialysis units*  *pmp *** | *Number of*  *beds in*  *dialysis units*  *pmp *** | *Current healthcare*  *expenditure per capita (€) **** |
| Piemonte | 0.9 | 1.0 | 212.4 | 178.2 | 1,889.1 |
| Valle d'Aosta | 1.6 | 1.7 | 265.1 | 241.7 | 1,810.1 |
| Lombardia | 1.4 | 1.0 | 196.7 | 184.0 | 1,874.4 |
| Trentino-Alto Adige | 1.4 | 0.7 | 158.5 | 159.7 | 1,736.8 |
| Veneto | 1.1 | 1.3 | 126.8 | 116.0 | 1,834.7 |
| Friuli-Venezia Giulia | 1.0 | 0.8 | 242.5 | 217.1 | 2,006.6 |
| Liguria | 0.7 | 0.7 | 220.2 | 160.1 | 1,951.9 |
| Emilia-Romagna | 1.6 | 1.1 | 170.9 | 169.7 | 2,010.1 |
| Toscana | 1.2 | 0.9 | 175.2 | 182.1 | 1,923.5 |
| Umbria | 1.4 | 1.0 | 215.2 | 207.4 | 1,874.6 |
| Marche | 1.4 | 1.3 | 204.6 | 195.5 | 1,822.7 |
| Lazio | 1.4 | 1.1 | 69.3 | 98.4 | 1,838.4 |
| Abruzzo | 2.0 | 1.3 | 103.8 | 172.2 | 1,762.1 |
| Molise | 0.6 | 1.6 | 210.9 | 319.6 | 1,944.0 |
| Campania | 1.7 | 1.6 | 75.8 | 125.8 | 1,669.6 |
| Puglia | 1.7 | 1.7 | 221.2 | 250.4 | 1,705.2 |
| Basilicata | 1.4 | 1.5 | 125.0 | 147.6 | 1,799.2 |
| Calabria | 1.0 | 1.9 | 212.9 | 208.9 | 1,678.5 |
| Sicilia | 1.7 | 1.7 | 86.9 | 108.5 | 1,697.5 |
| Sardegna | 1.9 | 1.5 | 277.2 | 326.2 | 1,746.3 |
| **** National Health Interview Survey (Istat 2013); ** Italian Society of Nephrology (SIN) Census 2014-15; *** Ministry of Economy and Finance (2012)*** | | | | | |

| **Supplementary Table S4. Net effect of the region as second-level variable: predicted post-estimation counts by region from models 1-4. Males** | | | | | | | | | | | | |
| --- | --- | --- | --- | --- | --- | --- | --- | --- | --- | --- | --- | --- |
|  | | | | | | | | | | | | |
| **Region** | **Model 1** | | | **Model 2** | | | **Model 3** | | | **Model 4** | | |
|  | **Estimate** | **95%CI** | **P** | **Estimate** | **95%CI** | **P** | **Estimate** | **95%CI** | **P** | **Estimate** | **95%CI** | **P** |
| Abruzzo | 0.00 | -0.11 ; 0.11 | 0.995 | -0.06 | -0.25 ; 0.13 | 0.552 | 0.07 | -0.08 ; 0.22 | 0.340 | 0.02 | -0.09 ; 0.13 | 0.785 |
| Basilicata | -0.01 | -0.15 ; 0.12 | 0.838 | -0.02 | -0.15 ; 0.11 | 0.772 | 0.03 | -0.11 ; 0.17 | 0.653 | -0.06 | -0.16 ; 0.03 | 0.199 |
| Calabria | 0.16 | 0.05 ; 0.27 | 0.004 | 0.19 | 0.05 ; 0.33 | 0.006 | -0.16 | -0.30 ; -0.01 | 0.037 | -0.09 | -0.19 ; 0.02 | 0.105 |
| Campania | 0.44 | 0.34 ; 0.54 | <0.001 | 0.41 | 0.29 ; 0.54 | <0.001 | 0.15 | 0.02 ; 0.27 | 0.018 | 0.04 | -0.04 ; 0.12 | 0.353 |
| Emilia-Romagna | -0.22 | -0.32 ; -0.11 | <0.001 | -0.24 | -0.35 ; -0.12 | <0.001 | -0.19 | -0.31 ; -0.06 | 0.003 | -0.08 | -0.18 ; 0.03 | 0.144 |
| Friuli-Venezia Giulia | -0.40 | -0.52 ; -0.27 | <0.001 | -0.36 | -0.51 ; -0.21 | <0.001 | -0.15 | -0.30 ; -0.01 | 0.040 | -0.12 | -0.22 ; -0.01 | 0.032 |
| Lazio | 0.19 | 0.09 ; 0.29 | <0.001 | 0.19 | 0.09 ; 0.29 | <0.001 | 0.20 | 0.08 ; 0.32 | 0.001 | -0.02 | -0.11 ; 0.08 | 0.744 |
| Liguria | 0.08 | -0.03 ; 0.19 | 0.162 | 0.14 | -0.05 ; 0.32 | 0.155 | 0.06 | -0.10 ; 0.22 | 0.484 | 0.08 | -0.03 ; 0.20 | 0.162 |
| Lombardia | -0.02 | -0.12 ; 0.07 | 0.622 | -0.03 | -0.12 ; 0.07 | 0.579 | 0.01 | -0.11 ; 0.13 | 0.905 | 0.01 | -0.08 ; 0.09 | 0.898 |
| Marche | -0.09 | -0.20 ; 0.03 | 0.132 | -0.09 | -0.21 ; 0.02 | 0.103 | -0.03 | -0.16 ; 0.10 | 0.629 | 0.03 | -0.07 ; 0.13 | 0.516 |
| Molise | 0.04 | -0.12 ; 0.19 | 0.651 | 0.10 | -0.13 ; 0.33 | 0.391 | 0.19 | 0.02 ; 0.36 | 0.031 | 0.08 | -0.03 ; 0.20 | 0.158 |
| Piemonte | -0.10 | -0.20 ; 0.001 | 0.052 | -0.06 | -0.21 ; 0.08 | 0.417 | -0.09 | -0.22 ; 0.04 | 0.189 | -0.05 | -0.17 ; 0.06 | 0.344 |
| Puglia | 0.17 | 0.07 ; 0.27 | 0.001 | 0.14 | 0.02 ; 0.27 | 0.022 | -0.12 | -0.24 ; 0.002 | 0.053 | 0.02 | -0.07 ; 0.12 | 0.624 |
| Sardegna | -0.11 | -0.22 ; 0.004 | 0.060 | -0.16 | -0.35 ; 0.02 | 0.078 | -0.07 | -0.22 ; 0.08 | 0.383 | 0.01 | -0.09 ; 0.11 | 0.823 |
| Sicilia | 0.42 | 0.33 ; 0.52 | <0.001 | 0.39 | 0.25 ; 0.52 | <0.001 | 0.13 | 0.01 ; 0.25 | 0.038 | 0.03 | -0.05 ; 0.11 | 0.515 |
| Toscana | 0.02 | -0.08 ; 0.12 | 0.711 | 0.03 | -0.07 ; 0.14 | 0.533 | 0.04 | -0.08 ; 0.16 | 0.504 | 0.02 | -0.07 ; 0.11 | 0.621 |
| Trentino-Alto Adige | -0.25 | -0.38 ; -0.12 | <0.001 | -0.25 | -0.38 ; -0.12 | <0.001 | -0.01 | -0.16 ; 0.14 | 0.902 | 0.03 | -0.07 ; 0.14 | 0.527 |
| Umbria | 0.01 | -0.11 ; 0.13 | 0.881 | 0.00 | -0.12 ; 0.12 | 0.989 | 0.04 | -0.09 ; 0.17 | 0.562 | 0.03 | -0.07 ; 0.12 | 0.597 |
| Valle d'Aosta | -0.21 | -0.45 ; 0.03 | 0.084 | -0.22 | -0.47 ; 0.02 | 0.068 | -0.02 | -0.21 ; 0.17 | 0.852 | 0.00 | -0.11 ; 0.11 | 0.991 |
| Veneto | -0.11 | -0.21 ; -0.01 | 0.030 | -0.09 | -0.21 ; 0.03 | 0.151 | -0.07 | -0.21 ; 0.06 | 0.300 | 0.02 | -0.09 ; 0.13 | 0.784 |

*Net-effect: regional differences around the national mean (average mortality), after adjusting for all the covariates considered at the first-level and second-level*

| **Supplementary Table S5. Net effect of the region as second-level variable: predicted post-estimation counts by region from models 1-4. Females.** | | | | | | | | | | | | |
| --- | --- | --- | --- | --- | --- | --- | --- | --- | --- | --- | --- | --- |
|  | | | | | | | | | | | | |
| **Region** | **Model 1** | | | **Model 2** | | | **Model 3** | | | **Model 4** | | |
|  | **Estimate** | **95%CI** | **P** | **Estimate** | **95%CI** | **P** | **Estimate** | **95%CI** | **P** | **Estimate** | **95%CI** | **P** |
| Abruzzo | -0.05 | -0.17 ; 0.08 | 0.459 | -0.05 | -0.16 ; 0.06 | 0.348 | 0.01 | -0.12 ; 0.14 | 0.920 | -0.04 | -0.18 ; 0.10 | 0.575 |
| Basilicata | 0.11 | -0.03 ; 0.25 | 0.128 | 0.04 | -0.09 ; 0.17 | 0.573 | 0.13 | -0.01 ; 0.27 | 0.066 | 0.04 | -0.07 ; 0.15 | 0.504 |
| Calabria | 0.15 | 0.03 ; 0.27 | 0.016 | -0.10 | -0.29 ; 0.09 | 0.303 | -0.23 | -0.36 ; -0.10 | 0.001 | -0.11 | -0.24 ; 0.02 | 0.095 |
| Campania | 0.49 | 0.38 ; 0.60 | <0.001 | 0.37 | 0.25 ; 0.49 | <0.001 | 0.13 | 0.01 ; 0.25 | 0.039 | 0.02 | -0.07 ; 0.12 | 0.641 |
| Emilia-Romagna | -0.13 | -0.24 ; -0.01 | 0.027 | -0.06 | -0.17 ; 0.04 | 0.248 | -0.15 | -0.27 ; -0.02 | 0.019 | -0.09 | -0.21 ; 0.04 | 0.167 |
| Friuli-Venezia Giulia | -0.44 | -0.58 ; -0.31 | <0.001 | -0.28 | -0.43 ; -0.12 | 0.001 | -0.15 | -0.31 ; 0.005 | 0.058 | -0.16 | -0.29 ; -0.03 | 0.018 |
| Lazio | 0.16 | 0.05 ; 0.28 | 0.004 | 0.23 | 0.13 ; 0.34 | <0.001 | 0.14 | 0.02 ; 0.26 | 0.021 | -0.06 | -0.18 ; 0.07 | 0.368 |
| Liguria | -0.02 | -0.14 ; 0.10 | 0.747 | 0.19 | 0.02 ; 0.36 | 0.031 | -0.01 | -0.16 ; 0.14 | 0.929 | 0.11 | -0.03 ; 0.24 | 0.118 |
| Lombardia | -0.06 | -0.17 ; 0.05 | 0.314 | 0.02 | -0.08 ; 0.13 | 0.683 | 0.04 | -0.08 ; 0.16 | 0.477 | 0.02 | -0.08 ; 0.13 | 0.646 |
| Marche | -0.07 | -0.19 ; 0.05 | 0.246 | -0.10 | -0.21 ; 0.01 | 0.064 | -0.02 | -0.15 ; 0.11 | 0.733 | 0.04 | -0.08 ; 0.17 | 0.509 |
| Molise | 0.02 | -0.14 ; 0.18 | 0.815 | -0.11 | -0.28 ; 0.05 | 0.188 | 0.18 | -0.004 ; 0.36 | 0.056 | 0.11 | -0.03 ; 0.24 | 0.116 |
| Piemonte | -0.24 | -0.36 ; -0.13 | <0.001 | -0.14 | -0.26 ; -0.02 | 0.018 | -0.13 | -0.25 ; -0.01 | 0.035 | -0.07 | -0.21 ; 0.07 | 0.344 |
| Puglia | 0.34 | 0.22 ; 0.45 | <0.001 | 0.17 | 0.03 ; 0.31 | 0.016 | -0.03 | -0.15 ; 0.09 | 0.588 | 0.05 | -0.08 ; 0.18 | 0.446 |
| Sardegna | 0.07 | -0.05 ; 0.19 | 0.266 | -0.02 | -0.14 ; 0.10 | 0.711 | 0.01 | -0.14 ; 0.17 | 0.880 | 0.04 | -0.08 ; 0.16 | 0.532 |
| Sicilia | 0.50 | 0.39 ; 0.62 | <0.001 | 0.35 | 0.22 ; 0.48 | <0.001 | 0.13 | 0.01 ; 0.25 | 0.029 | 0.04 | -0.06 ; 0.13 | 0.455 |
| Toscana | -0.06 | -0.18 ; 0.05 | 0.295 | 0.06 | -0.07 ; 0.18 | 0.365 | 0.05 | -0.07 ; 0.17 | 0.445 | 0.02 | -0.09 ; 0.12 | 0.764 |
| Trentino-Alto Adige | -0.20 | -0.33 ; -0.06 | 0.006 | 0.02 | -0.17 ; 0.20 | 0.850 | 0.08 | -0.09 ; 0.25 | 0.355 | 0.09 | -0.04 ; 0.22 | 0.179 |
| Umbria | -0.05 | -0.18 ; 0.08 | 0.416 | 0.02 | -0.10 ; 0.15 | 0.709 | 0.04 | -0.09 ; 0.17 | 0.534 | 0.03 | -0.08 ; 0.13 | 0.622 |
| Valle d'Aosta | -0.33 | -0.59 ; -0.08 | 0.009 | -0.42 | -0.67 ; -0.17 | 0.001 | -0.10 | -0.30 ; 0.09 | 0.311 | -0.04 | -0.18 ; 0.11 | 0.616 |
| Veneto | -0.17 | -0.28 ; -0.06 | 0.004 | -0.18 | -0.27 ; -0.08 | <0.001 | -0.11 | -0.24 ; 0.01 | 0.076 | -0.04 | -0.18 ; 0.10 | 0.573 |

*Net-effect: regional differences around the national mean (average mortality), after adjusting for all the covariates considered at the first-level and second-level*

| **Supplementary Table S6 – Results of preliminary analysis to select nephrology offer variables for multivevel models** | | | | | | | | |
| --- | --- | --- | --- | --- | --- | --- | --- | --- |
|  | | | | | | | | |
|  | | **MALES** | | |  | **FEMALES** | | |
| **S6-a - Model with individual covariates and number of nurses in dialysys units** | | | | | | | | |
| **VARIABLES** | | RR | 95%CI | P |  | RR | 95%CI | P |
| **Education level** | Elementary school or less | 1.54 | 1.48-1.60 | <0.001 |  | 1.92 | 1.85-2.00 | <0.001 |
|  | Middle school | 1.27 | 1.23-1.31 | <0.001 |  | 1.55 | 1.49-1.61 | <0.001 |
|  | High school diploma | 1.12 | 1.08-1.16 | <0.001 |  | 1.22 | 1.16-1.27 | <0.001 |
|  | University Degree or more | 1 | - | - |  | 1 | - | - |
| **Age class** | 30-64 | 1 | - | - |  | 1 | - | - |
|  | 65-74 | 7.95 | 7.51-8.42 | <0.001 |  | 7.36 | 6.85-7.91 | <0.001 |
|  | 75-84 | 34.17 | 32.45-35.99 | <0.001 |  | 32.15 | 30.14-34.30 | <0.001 |
|  | 85+ | 153.36 | 145.69-161.44 | <0.001 |  | 153.01 | 143.63-163.01 | <0.001 |
| **Citizenship** | Italian | 1 | - | - |  | 1 | - | - |
|  | Highly Developed Countries | 0.40 | 0.27-0.58 | <0.001 |  | 0.37 | 0.30-0.46 | <0.001 |
|  | High Migratory Pressure Countries | 0.37 | 0.30-0.45 | <0.001 |  | 0.58 | 0.41-0.83 | 0.003 |
| **Number of nurses in dialysys units** | Q1 (<126) | 1.37 | 1.11-1.68 | 0.003 |  | 1.38 | 1.08-1.77 | 0.010 |
|  | Q2 (126-200) | 0.98 | 0.79-1.20 | 0.831 |  | 0.95 | 0.75-1.22 | 0.705 |
|  | Q3 (201-216) | 1.10 | 0.90-1.36 | 0.356 |  | 1.03 | 0.81-1.33 | 0.787 |
|  | Q4 (>216) | 1 | - | - |  | 1 | - | - |
| **S6-b - Model with individual covariates and number of beds in dialysys units** | | | | | | | | |
| **VARIABLES** | | RR | 95%CI | P |  | RR | 95%CI | P |
| **Education level** | Elementary school or less | 1.54 | 1.48-1.60 | <0.001 |  | 1.92 | 1.85-2.00 | <0.001 |
|  | Middle school | 1.27 | 1.23-1.31 | <0.001 |  | 1.55 | 1.49-1.61 | <0.001 |
|  | High school diploma | 1.12 | 1.08-1.16 | <0.001 |  | 1.22 | 1.16-1.27 | <0.001 |
|  | University Degree or more | 1 | - | - |  | 1 | - | - |
| **Age class** | 30-64 | 1 | - | - |  | 1 | - | - |
|  | 65-74 | 7.95 | 7.51-8.42 | <0.001 |  | 7.36 | 6.85-7.91 | <0.001 |
|  | 75-84 | 34.17 | 32.45-35.99 | <0.001 |  | 32.15 | 30.14-34.30 | <0.001 |
|  | 85+ | 153.36 | 145.69-161.44 | <0.001 |  | 153.01 | 143.63-163.01 | <0.001 |
| **Citizenship** | Italian | 1 | - | - |  | 1 | - | - |
|  | Highly Developed Countries | 0.40 | 0.27-0.58 | <0.001 |  | 0.37 | 0.30-0.46 | <0.001 |
|  | High Migratory Pressure Countries | 0.37 | 0.30-0.45 | <0.001 |  | 0.58 | 0.41-0.83 | 0.003 |
| **Number of beds in dialysis units** | Q1 (<157) | 1.35 | 1.09-1.69 | 0.0072 |  | 1.34 | 1.04-1.74 | 0.026 |
|  | Q2 (157-179) | 1.01 | 0.81-1.26 | 0.9288 |  | 0.94 | 0.73-1.22 | 0.662 |
|  | Q3 (180-210) | 1.13 | 0.91-1.41 | 0.2621 |  | 1.05 | 0.81-1.36 | 0.696 |
|  | Q4 (>210) | 1 | - | - |  | 1 | - | - |
| **S6-c - Model with individual covariates and number of doctors in dialysys units** | | | | | | | | |
| **VARIABLES** | | RR | 95%CI | P |  | RR | 95%CI | P |
| **Education level** | Elementary school or less | 1.54 | 1.48-1.60 | <0.001 |  | 1.92 | 1.85-2.00 | <0.001 |
|  | Middle school | 1.27 | 1.23-1.31 | <0.001 |  | 1.55 | 1.49-1.61 | <0.001 |
|  | High school diploma | 1.12 | 1.08-1.16 | <0.001 |  | 1.22 | 1.16-1.27 | <0.001 |
|  | University Degree or more | 1 | - | - |  | 1 | - | - |
| **Age class** | 30-64 | 1 | - | - |  | 1 | - | - |
|  | 65-74 | 7.95 | 7.51-8.42 | <0.001 |  | 7.36 | 6.85 | <0.001 |
|  | 75-84 | 34.17 | 32.45-35.99 | <0.001 |  | 32.15 | 30.14 | <0.001 |
|  | 85+ | 153.37 | 145.70-161.45 | <0.001 |  | 153.02 | 143.63 | <0.001 |
| **Citizenship** | Italian | 1 | - | - |  | 1 | - | - |
|  | Highly Developed Countries | 0.40 | 0.27-0.58 | <0.001 |  | 0.37 | 0.30-0.46 | <0.001 |
|  | High Migratory Pressure Countries | 0.37 | 0.30-0.45 | <0.001 |  | 0.58 | 0.41-0.83 | 0.003 |
| **Number of doctors in dialysis units** | Q1 (<47) | 1.04 | 0.81-1.35 | 0.738 |  | 1.03 | 0.78-1.36 | 0.846 |
|  | Q2 (47-56) | 1.03 | 0.80-1.33 | 0.811 |  | 0.99 | 0.75-1.31 | 0.937 |
|  | Q3 (57-65) | 0.89 | 0.69-1.15 | 0.386 |  | 0.80 | 0.60-1.06 | 0.118 |
|  | Q4 (>66+) | 1 | - | - |  | 1 | - | - |
| **S6-d - Model with individual covariates and number of hospital admissions in nephrology departments** | | | | | | | | |
| **VARIABLES** | | RR | 95%CI | P |  | RR | 95%CI | P |
| **Education level** | Elementary school or less | 1.54 | 1.48-1.60 | <0.001 |  | 1.92 | 1.85-2.00 | <0.001 |
|  | Middle school | 1.27 | 1.23-1.31 | <0.001 |  | 1.55 | 1.49-1.61 | <0.001 |
|  | High school diploma | 1.12 | 1.08-1.16 | <0.001 |  | 1.22 | 1.16-1.27 | <0.001 |
|  | University Degree or more | 1 | - | - |  | 1 | - | - |
| **Age class** | 30-64 | 1 | - | - |  | 1 | - | - |
|  | 65-74 | 7.95 | 7.51-8.42 | <0.001 |  | 7.36 | 6.85-7.91 | <0.001 |
|  | 75-84 | 34.17 | 32.45-35.99 | <0.001 |  | 32.15 | 30.14-34.30 | <0.001 |
|  | 85+ | 153.36 | 145.69-161.43 | <0.001 |  | 153.01 | 143.63-163.01 | <0.001 |
| **Citizenship** | Italian | 1 | - | - |  | 1 | - | - |
|  | Highly Developed Countries | 0.40 | 0.27-0.58 | <0.001 |  | 0.37 | 0.30-0.46 | <0.001 |
|  | High Migratory Pressure Countries | 0.37 | 0.30-0.45 | <0.001 |  | 0.58 | 0.41-0.83 | 0.003 |
| **Number of hospital admissions in nephrology departments** | Q1 (960) | 1.25 | 0.98-1.61 | 0.073 |  | 1.28 | 0.96-1.71 | 0.091 |
|  | Q2 (960-1317) | 1.07 | 0.83-1.37 | 0.611 |  | 1.06 | 0.80-1.41 | 0.689 |
|  | Q3 (1318-1577) | 1.09 | 0.86-1.40 | 0.470 |  | 1.15 | 0.87-1.54 | 0.329 |
|  | Q4 (>1577) | 1 | - | - |  | 1 | - | - |
| **S6-e - Model with individual covariates and number of beds in nephrology departments** | | | | | | | | |
| **VARIABLES** | | RR | 95%CI | P |  | RR | 95%CI | P |
| **Education level** | Elementary school or less | 1.54 | 1.48-1.60 | <0.001 |  | 1.92 | 1.85-2.00 | <0.001 |
|  | Middle school | 1.27 | 1.23-1.31 | <0.001 |  | 1.55 | 1.49-1.61 | <0.001 |
|  | High school diploma | 1.12 | 1.08-1.16 | <0.001 |  | 1.22 | 1.16-1.27 | <0.001 |
|  | University Degree or more | 1 | - | - |  | 1 | - | - |
| **Age class** | 30-64 | 1 | - | - |  | 1 | - | - |
|  | 65-74 | 7.95 | 7.51-8.42 | <0.001 |  | 7.36 | 6.85-7.91 | <0.001 |
|  | 75-84 | 34.17 | 32.45-35.99 | <0.001 |  | 32.15 | 30.14-34.29 | <0.001 |
|  | 85+ | 153.35 | 145.68-161.43 | <0.001 |  | 153 | 143.62-163.00 | <0.001 |
| **Citizenship** | Italian | 1 | - | - |  | 1 | - | - |
|  | Highly Developed Countries | 0.40 | 0.27-0.58 | <0.001 |  | 0.37 | 0.30-0.46 | <0.001 |
|  | High Migratory Pressure Countries | 0.37 | 0.30-0.45 | <0.001 |  | 0.58 | 0.41-0.83 | 0.003 |
| **Number of beds in nephrology departments** | Q1 (<25) | 1.13 | 0.89-1.43 | 0.315 |  | 1.16 | 0.87-1.54 | 0.308 |
|  | Q2 (25-34) | 0.89 | 0.70-1.13 | 0.335 |  | 0.91 | 0.68-1.20 | 0.493 |
|  | Q3 (35-46) | 0.97 | 0.76-1.23 | 0.780 |  | 1 | 0.75-1.33 | 0.994 |
|  | Q4 (>46) | 1 | - | - |  | 1 | - | - |

| **Supplementary Table S7 – Description of the structure of multilevel models** | | | | | | |
| --- | --- | --- | --- | --- | --- | --- |
| **Model** | **1^st^ Level Covariates (Individual)** | | | **2^nd^ Level Covariates (Regional)** | | |
| 1 | education level | age class | citizenship | - | - | - |
| 2 | education level | age class | citizenship | prevalence of CKD | - | - |
| 3 | education level | age class | citizenship | prevalence of CKD | healt care expenditure | - |
| 4 | education level | age class | citizenship | prevalence of CKD | healt care expenditure | number of nurses in dialysis |


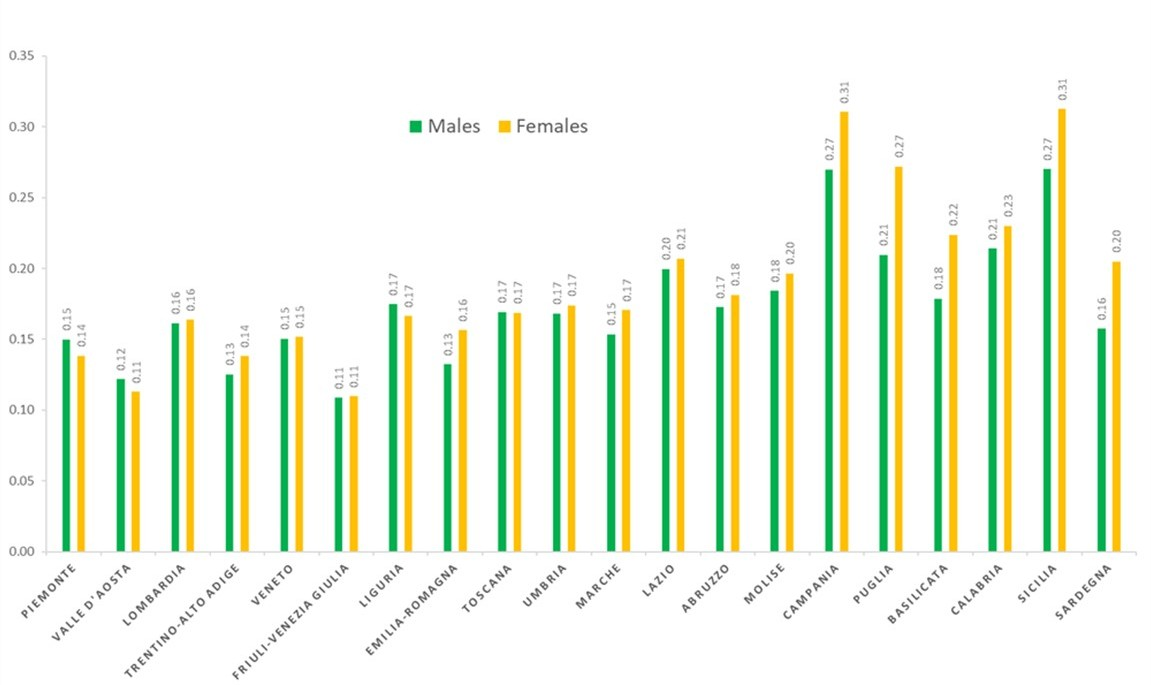


**Supplementary Figure S1. Age standardized renal mortality rates (*1000 person-years) by region of residence and sex.**

*Northern regions: Piemonte, Valle d’Aosta, Lombardia, Trentino-Alto Adige, Veneto, Friuli-Venezia Giulia, Liguria, Emilia-Romagna*

*Central regions: Toscana, Umbria, Marche, Lazio, Abruzzo*

*Southern and Island regions: Molise, Campania, Puglia, Basilicata, Calabria, Sicilia, Sardegna*
